# Structural, Stability, Dynamic and Binding Properties of the ALS-Causing T46I Mutant of the hVAPB MSP Domain as Revealed by NMR and MD Simulations

**DOI:** 10.1371/journal.pone.0027072

**Published:** 2011-11-01

**Authors:** Shixiong Lua, Haina Qin, Liangzhong Lim, Jiahai Shi, Garvita Gupta, Jianxing Song

**Affiliations:** 1 Department of Biological Sciences, Faculty of Science, National University of Singapore, Singapore, Singapore; 2 Department of Biochemistry, Yong Loo Lin School of Medicine, National University of Singapore, Singapore, Singapore; University of Rome, Italy

## Abstract

T46I is the second mutation on the hVAPB MSP domain which was recently identified from non-Brazilian kindred to cause a familial amyotrophic lateral sclerosis (ALS). Here using CD, NMR and molecular dynamics (MD) simulations, we characterized the structure, stability, dynamics and binding capacity of the T46I-MSP domain. The results reveal: 1) unlike P56S which we previously showed to completely eliminate the native MSP structure, T46I leads to no significant disruption of the native secondary and tertiary structures, as evidenced from its far-UV CD spectrum, as well as Cα and Cβ NMR chemical shifts. 2) Nevertheless, T46I does result in a reduced thermodynamic stability and loss of the cooperative urea-unfolding transition. As such, the T46I-MSP domain is more prone to aggregation than WT at high protein concentrations and temperatures *in vitro*, which may become more severe in the crowded cellular environments. 3) T46I only causes a 3-fold affinity reduction to the Nir2 peptide, but a significant elimination of its binding to EphA4. 4) EphA4 and Nir2 peptide appear to have overlapped binding interfaces on the MSP domain, which strongly implies that two signaling networks may have a functional interplay *in vivo*. 5) As explored by both H/D exchange and MD simulations, the MSP domain is very dynamic, with most loop residues and many residues on secondary structures highly fluctuated or/and exposed to bulk solvent. Although T46I does not alter overall dynamics, it does trigger increased dynamics of several local regions of the MSP domain which are implicated in binding to EphA4 and Nir2 peptide. Our study provides the structural and dynamic understanding of the T46I-causing ALS; and strongly highlights the possibility that the interplay of two signaling networks mediated by the FFAT-containing proteins and Eph receptors may play a key role in ALS pathogenesis.

## Introduction

Amyotrophic lateral sclerosis (ALS) is the most prevalent fatal motor neuron disease, which affects people of all race and ethnic background. Approximately 10% of the cases have hereditary background while the rest are sporadic. Seven ALS-causative genes have been identified to date but the most characterized is ALS1 encoding mutants of Cu/Zn-superoxide dismutase (SOD1). So far, despite intense studies, the exact mechanism underlying ALS pathogenesis remains poorly understood and there is no efficient therapy for ALS. As sporadic and familial ALS affects the same neurons with similar pathology, it is believed that knowledge and therapeutic approaches/agents developed on mutant models can be translated to sporadic ALS [1], [2].

Recently, ALS8 was identified from a large Brazilian family with dominant motor neuron diseases, which encodes a mutated VAPB (vesicle-associated membrane protein-associated protein B). The missense P56S point mutation results in a typical ALS phenotype with rapid progression or late onset spinal muscular atrophy [2]–[5]. Very recently, another mutation T46I on VAPB has been unraveled to also cause ALS in non-Brazilian kindred [6]. The human VAP family proteins were originally identified as homologues of vesicle-associated membrane protein (VAMP)-associated protein (VAP) in *Aplysia californica* (aVAP33) [7], [8], which include VAPA, VAPB, and VAPC. VAPA and VAPB share ∼60% sequence identity while VAPB and VAPC are alternatively spliced variants. VAP proteins are ubiquitously expressed, type II integral membrane proteins that localize to the endoplasmic reticulum (ER) and pre-Golgi intermediates [9], which have been implicated in the regulation of neurotransmitter release, ER-Golgi and intra-Golgi transport [10], [11]. Moreover, VAP proteins have been shown to target lipid-binding proteins carrying a short motif with two phenylalanines in an acidic tract (FFAT motif) to the ER [12], [13]. The FFAT motif with the consensus amino acid sequence EFFDAxE was conserved in several lipid-binding protein families involved in the transfer of lipids between the ER and other organelles [14]–[18]. The VAP proteins also interact with intracellular proteins [19], [20] including Nir1, Nir2, and Nir3 via the FFAT motif which differentially affects the organization of the ER [11].

Despite owning very diverse functions, VAPA and VAPB proteins are composed of three conserved domains, namely an N-terminal immunoglobulin-like β sheet domain with 22% sequence identity to the major sperm protein (MSP), a central coiled-coil domain, and a C-terminal transmembrane domain. After the P56S mutation was identified to be associated to ALS, the VAPB-MSP domain received intensive studies regarding its functions and the biological consequence of the mutation [21]–[28]. The P56S mutation results in severe aggregation and alteration of unfolded protein response (UPR) in ER [6], [21]–[28]. Strikingly, the cleaved VAPB MSP domain was recently found to act as a novel ligand class for Eph receptors [29]. The connection of VAPB to Eph receptors may shed critical light on the core signaling networks involved in ALS pathogenesis and also represent a promising target for therapeutic interventions [30]. Indeed, Eph receptors constitute the largest family of tyrosine kinases, with 16 members in mammals and chick divided into two classes, EphA and EphB, which are respectively activated by six glycosylphosphatidylinositol-anchored ephrin-A ligands or three transmembrane ephrin-B ligands to mediate an extremely wide spectrum of biological functions through signals that are generated by both receptor and ligand activation [31].

Previously, we have determined the crystal structure of the hVAPB MSP domain, which adopts a well-defined MSP fold with a cooperative urea-unfolding transition [32]. Moreover, with our discovery that insoluble proteins could in fact be solubilized in salt-free water [33]–[35], we have succeeded in characterizing the P56S mutant by NMR spectroscopy and demonstrated that the P56S mutation completely eliminates the native MSP structure and binding capacity. In particular, the unstructured P56S mutant becomes severely aggregated in buffers mimicking the physiological conditions [32].

In the present study, we aimed to characterize the newly-identified T46I mutant. By extensive CD, NMR characterization and molecular dynamics (MD) simulations, we found that the T46I-MSP domain still retains the native MSP structure with overall dynamics largely unaltered. Nevertheless the T46I mutation on the MSP domain does lead to a reduction of its thermodynamic stability and loss of the cooperative urea-unfolding transition. As such, the T46I-MSP domain is much more prone to aggregation at high protein concentrations and temperatures. Noticeably, the T46I mutation results in only a 3-fold reduction of its binding affinity to the Nir2 peptide, but a significant abolishment of the binding ability to EphA4.

## Methods

### Cloning, expression, and purification of the WT and T46I MSP domains

The DNA fragment encoding the wild-type (WT) hVAPB MSP domain (residues 1–125) was cloned into a modified pET32a vector (Novagen) as previously described [32]. The T46I mutation on the MSP domain was generated by use of site-directed mutagenesis [36]. The vectors were transformed into *E. coli* BL21 (DE3) cells (Novagen) for protein expression. Most WT and a small portion of T46I proteins were found in supernatant and thus were first purified by Ni^2+^-affinity chromatography (Qiagen) under the native condition, followed by in-gel cleavage to remove the His-tag. The released WT and T46I proteins were further purified on an AKTA FPLC machine (Amersham Biosciences) using a gel filtration column (HiLoad 16/60 Superdex 200), followed by an anion-exchange column (Mono Q 5/50). The generation and purification of the EphA4 ligand-binding domain (181 residues) followed the protocol we previously established [37].

The production of the isotope-labeled MSP and EphA4 proteins for NMR studies followed a similar procedure except that the bacteria were grown in M9 medium with the addition of [^15^NH_4_]_2_SO_4_ for ^15^N labeling and [^15^NH_4_]_2_SO_4_/[^13^C]-glucose for ^15^N-/^13^C double labeling [32], [37]. The purity of all protein samples was checked by the SDS-PAGE gel and their molecular weights were verified by a Voyager STR matrix-assisted laser desorption ionization time-of-flight-mass spectrometer (Applied Biosystems). The concentration of protein samples was determined by the spectroscopic method in the presence of denaturant [38].

### Structure and thermodynamic stability as measured by Circular Dichroism [CD]

All CD experiments were carried out in a Jasco J-810 spectropolarimeter (Jasco Corporation, Tokyo, Japan) as previously described [32]–[35]. The protein concentration is 20 µM for all far-UV CD experiments. To measure the thermodynamic stability of the T46I-MSP domain, a series of far-UV CD spectra were collected by varying the urea concentrations from 0 to 8 M and subsequently the unfolding curve was constructed by plotting the ellipiticity values at 222 nm versus the molar concentration of urea [32].

### ITC characterization of binding activity

Isothermal titration calorimetry (ITC) experiments were performed using a Microcal VP machine as previously described [35]. The Nir2 peptide (EEEFFDAHE) was purchased from Genesis Biotech Inc. and further purified by HPLC on a reverse-phase C_18_ column. Titrations were conducted in the buffer (50 mM Tris-HCl, 150 mM NaCl, 2 mM DTT, pH 7.5) at 25°C. The peptide or EphA4 (300 µM) was loaded into the syringe while the wild-type hVAPB MSP protein (10 µM) was placed in the sample cell. To obtain thermodynamic binding parameters, the titration data after subtracting the blank values were fitted to a single binding site model using the built-in software ORIGIN version 5.0 (Microcal Software Inc.).

### NMR experiments

The T46I-MSP sample was prepared in 10 mM phosphate buffer, pH 6.8. All NMR data were collected at 25°C on an 800-MHz Bruker Avance spectrometer equipped with a shielded cryoprobe as described before [32], [35]. For HSQC characterization, samples were prepared at a protein concentration of 100 µM while for achieving sequential assignments, triple-resonance experiments including HNCACB and CBCA(CO)NH were acquired on a double-labeled sample at a protein concentration of ∼200 µM. The spectral processing and analysis was performed by using the program DANGLE in CCPNMR [39].

To conduct hydrogen-deuterium (H/D) exchange experiments, the ^15^N-labeled WT- or T46I-MSP domains in the 10 mM (pH 6.8) phosphate buffer was lyophilized and then re-dissolved in D_2_O. Progress of the exchange process between amide protons and deuterium was followed by collecting a series of successive HSQC spectra starting immediately after the sample re-solubilization in D_2_O. All exchange experiments were conducted on an 800 MHz Bruker Avance spectrometer at 25°C. The first HSQC spectrum was collected after 15 min, and the last spectra were acquired after 2.5 h.

For NMR HSQC characterization of the binding interactions of WT-/T46I-MSP to the Nir2 peptide; as well as to the EphA4 ligand-binding domain, two-dimensional ^1^H-^15^N HSQC spectra of the ^15^N-labeled WT-/T46I-MSP domains were acquired at a protein concentration of 100 µM in the absence or presence of the Nir2 peptide, or EphA4 at different molar ratios, including 1∶0.5; 1∶1; 1∶2; 1∶4; 1∶6; and 1∶8. Similarly, the binding interactions were also visualized by collecting HSQC spectra of the ^15^N-labeled EphA4 ligand-binding domain at a protein concentration of 100 µM in the absence or presence of WT- or T46I-MSP domain at the same molar ratios. By superimposing the HSQC spectra, the shifted or disappeared HSQC peaks could be identified and further assigned to the corresponding residues of the WT-/T46I-MSP domains or EphA4 ligand-binding domain whose assignment was previously achieved [37], [40], [41].

### Molecular docking of the MSP-EphA4 complex

The models of the MSP-EphA4 complex were constructed as we previously conducted on other systems [37], [41], by use of the HADDOCK software [42] in combination with CNS [43], which makes use of chemical shift perturbation data to derive the docking while allowing various degrees of flexibility. The docking procedure was performed by three steps as follows: first, randomization and rigid body energy minimization; second, semi-flexible simulated annealing; and third, flexible explicit solvent refinement. All MSP and EphA4 residues with HSQC peaks disappeared and significantly shifted were set to be “active” residues, whereas neighbors of active residues were defined as “passive” residues according to HADDOCK definition. One thousand structures were generated during the rigid body docking, and the best 50 structures were selected for semi-flexible simulated annealing, followed by water refinement. 2 structures with the lowest energies were selected for detailed analysis and display.

### Molecular dynamics (MD) simulations

To unravel the dynamic behaviors of both WT- and T46I-MSP domains, two independent, 15-ns MD simulations were performed for each of them. The WT-MSP structure used for MD simulations was 3IKK we previously determined [35]. As our current experimental results clearly indicate that the T46I-MSP domain still has the structure highly similar to the WT-MSP domain, the initial T46I structure was generated by using Mutagenesis command in pymol (www.pymol.org) to mutate the Thr46 residue to Ile with the option of backbone-dependent rotamers. Out of 5 Ile rotamers, we picked the one that has the highest representation of 81.9%, which has the least steric hindrance. This initial T46I structure was subsequently subjected to a 5000-step energy minimization using steepest descent algorithm and position restraint equilibration for 200 ps.

Simulations for both the WT- and T46I-MSP domains were set up as previously described [36]. Briefly, the simulation cell is a periodic cubic box with a minimum distance of 9 Å between the protein and the box walls to ensure the proteins would not directly interact with its own periodic image. The water molecules, described using the TIP3P model, were filled in the periodic cubic box for the all atom simulation. Each set of MD simulations was implemented by using the program GROMACS [44] for 15 ns, with the AMBER 99SB-ILDN all-atom force field [45]. The long-range electrostatic interactions were treated using the fast particle-mesh Ewald summation method [46]. The temperature during simulations was kept constant at 300 K by Berendsen's coupling. The pressure was held at 1 bar. The isothermal compressibility was 4.6*10^−5^ bar^−1^. The time step was set as 2 fs. All bond lengths including hydrogen atoms were constrained by the LINCS algorithm [47]. Prior to MD simulations, all the initial structures were relaxed by 5000 steps of energy minimization using steepest descent algorithm, followed by position restraint equilibration for 200 ps.

## Results

### Structure and thermodynamic stability of the T46I-MSP domain

**Figure 1:**
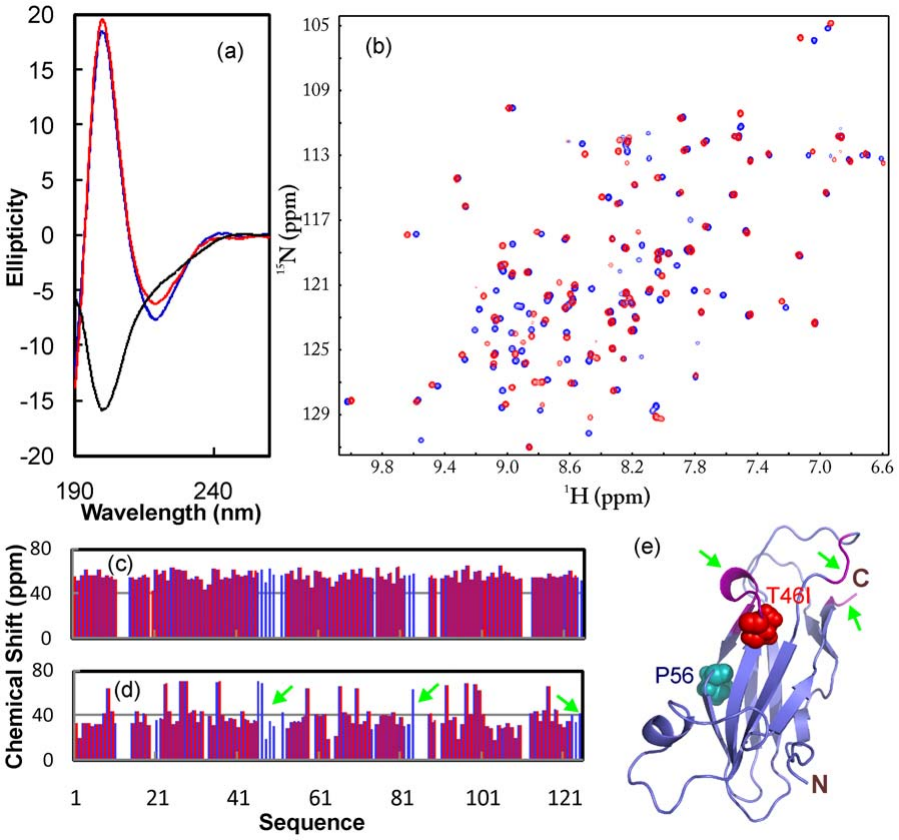
Structural characterization of the T46I-MSP domain. (a). Far UV CD spectra of the WT- (blue), T46I- (red) and P56S- (black) MSP domains at protein concentrations of 20 µM. (b). Superimposition of the two-dimensional ^1^H-^15^N NMR HSQC spectra of the WT- (blue) and T46I- (red) MSP domains at protein concentrations of 100 µM at pH 6.8. Cα (c) and Cβ (d) chemical shifts of the WT- (blue) and T46I- (red) MSP domains. The residues with the disappeared HSQC peaks are indicated by green arrows. (e). The crystal structure of the WT-MSP domain we previously determined (32) to which the T46I residues with their HSQC peaks undetected were mapped. The two mutation residues causing ALS, P56S and T46I, were displayed as spheres.

In the present study, by using site-directed mutagenesis we constructed the T46I-MSP domain by replacing Thr46 with Ile on the WT-MSP domain we previously generated [32]. Subsequently the T46I-MSP domain was expressed in *E. coli* BL21 cells. Although a large portion of the T46I protein existed in inclusion body, there was a small portion of the soluble T46I form in supernatant. Consequently we purified the soluble T46I protein by Ni^2+^-affinity column under native condition, followed by FPLC ion-exchange chromatography. The purified T46I domain with His-tag removed has a far-UV CD spectrum highly similar to that of WT (Figure 1a). This strongly indicates that T46I has a native-like secondary structure. Furthermore, the T46I-MSP domain also has a well-dispersed HSQC spectrum (Figure 1b) in which many peaks are even superimposable to those of WT, suggesting that T46I is also well-folded. Despite the aggregation of T46I at protein concentrations higher >300 µM, we managed to collect a pair of triple-resonance NMR spectra, namely HNCACB and CBCA(CO)NH, on a double-labeled sample at a protein concentration of ∼200 µM, which thus allowed our successful sequential assignment. Although HSQC peaks of three regions were not detectable for T46I, including residues Ile46-Tyr52, Glu82-Ser84 and C-terminal Ph123-Leu125, Cα and Cβ chemical shifts of the remaining T46I residues are almost identical to those of WT (Figures 1c, 1d and 1e). These results provide the strongest evidence that the T46I-MSP domain assumes a three-dimensional structure highly similar to that of WT.

**Figure 2:**
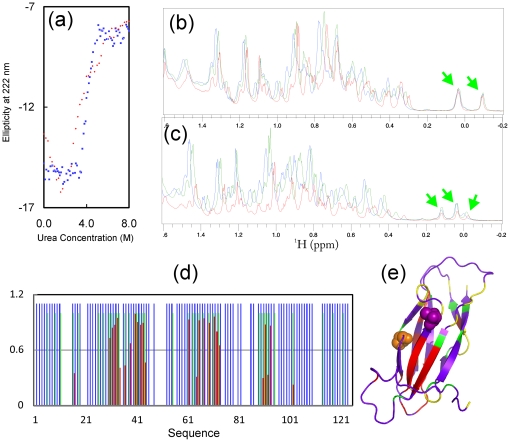
Stability of the T46I-MSP domain. (a). The urea-induced unfolding curves of the WT- (blue) and T46I- (red) MSP domains as reflected by changes of the ellipticity at 222 nm with urea concentrations ranging from 0 to 8 M. One-dimensional NMR spectra of the WT- (b) and T46I- (c) MSP domains at 15°C (green) 25°C (blue) and 45°C (red). (d). Hydrogen-deuterium (H/D) exchange results for the WT-MSP domain. Blue bars: residues with HSQC peaks detectable in the 10 mM phosphate buffer at pH 6.8. Green bars: residues with HSQC peaks detectable 15 min after re-dissolving the lyophilized protein powder in D_2_O. Red bars: residues with HSQC peaks detectable 2.5 hr after re-dissolving the lyophilized protein powder in D_2_O. (e). The crystal structure of the MSP domain with the H/D exchange results mapped. The yellow is to indicate the residues undetectable even in the 10 mM phosphate buffer at pH 6.8.

Thermodynamic stability is an important factor mediating protein aggregation particularly in the crowding cellular environments [48]. Here we assessed the thermodynamic stability of T46I by use of the urea-unfolding method previously for WT [32]. Markedly, unlike WT with a cooperative unfolding transition, T46I shows a non-cooperative transition as well as reduced stability (Figure 2a). T46I starts unfolding at a urea concentration of ∼1.6 M and shows no ending even at 8 M, while WT starts at ∼3.5 M and ends at ∼5.1 M. As a result of losing the cooperative unfolding transition, the precise energy of thermodynamic stability could not be fitted out from the T46I unfolding curve.

The loss of a cooperative unfolding transition of T46I implies that its tight tertiary packing is disrupted to some degree [49], [50] and consequently the T46I molecule may be more accessible to partially- or highly unfolded states prone to aggregation. Indeed, we observed that aggregation occurred for T46I at high protein concentrations and temperatures. For example, while at 45°C the WT sample showed no precipitation and has almost no change of the upfield peaks in the one-dimensional NMR proton spectra (Figure 2b), a large portion of the T46I protein precipitated at ∼42°C and the remaining protein has a dramatic structural alteration as evidenced from the changes of the upfield peaks (Figure 2c).

The high tendency of T46I to aggregate prevented from further investigations on its dynamics on ps-ns and µs-ms time scales by NMR relaxation measurements which require a sample with a much higher concentration for collecting high-quality data. Therefore, here we utilized NMR hydrogen/deuterium (H/D) exchange and to assess the backbone dynamics for both WT and T46I on minute-hour time scale. As well-established, in solution labile hydrogens such as amide protons on proteins are continually exchanging with the solvent at different rates, depending on a variety of factors associated with their environment including their exposure to the solvent or their involvement in H-bonds. Consequently, amide H/D exchange experiments offer a sensitive reflection of the exposure degree of amide protons to the solvent [51]. Surprisingly, as seen in Figure 2d, even for WT in the buffer, there are ∼12% of 116 non-proline residues whose HSQC peaks were not detectable. Upon subjecting to H/D exchange, ∼66% of the total residues have completely exchanged with deuterium within the experimental dead time (15 min). These fast-exchange rate residues cover not only most residues on the non-secondary structure regions, but also on the β-strands and helical fragment (Figure 2e). After 2.5 h, amide protons of more residues exchanged and consequently only ∼22% of the total residues have persisted HSQC peaks, which were characterized as slow-exchange-rate residues (Figures 2d and 2e). These results reveal that the hVAPB MSP domain is very dynamic on the min-hr time scale, similar to what we observed on the human ephrin-B2 [52]. Moreover, we have also characterized the amide H/D exchanges for T46I and no significant difference was found (data not shown), suggesting that T46I has a largely unchanged dynamics on the min-hr time scale.

### Interactions of WT-/T46I-MSP domains to Nir2 peptide

**Figure 3:**
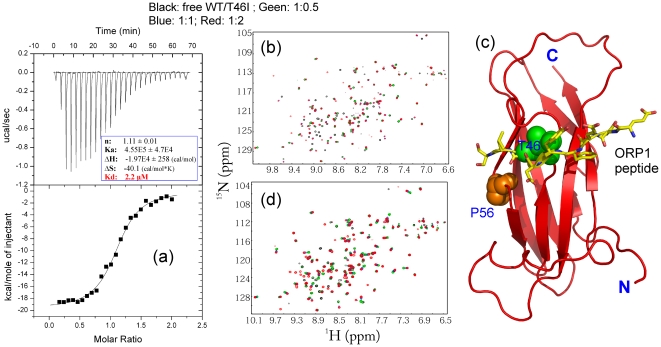
Interaction between the T46I-MSP domain and the Nir2 peptide. (a). The ITC titration profile of the binding reaction of the T46I-MSP domain to the Nir2 peptide [upper panel]; and integrated values for reaction heats with subtraction of the corresponding blank results normalized by the amount of ligand injected versus molar ratio of MSP/Nir2 (lower panel). Thermodynamic binding parameters obtained from fitting the data are shown in the box. Superimposition of the two-dimensional ^1^H-^15^N NMR HSQC spectra of the WT- (b) and T46I- (d) MSP domains in the absence (black) and presence of the Nir2 peptide at molar ratios 1∶0.5 (green), 1∶1 (blue) and 1∶2 (red). (c). Crystal structure of the VAPA MSP-ORP1 complex to illustrate the MSP-peptide binding interface [12].

The VAPB MSP domain was demonstrated to functionally interact with the FFAT-motif containing Nir2 protein [11] and previously we measured the dissociation constant (Kd) between the WT-MSP domain and a Nir2 peptide to be 0.65 µM by ITC [32]. Here we assessed the binding of T46I to the same peptide under the same conditions. The ITC result shows that T46I is still able to bind the peptide but the affinity has a 3-fold reduction, with a Kd of 2.2 µM (Figure 3a).

On the other hand, we also used HSQC titrations to visualize the binding of the WT- and T46I-MSP domains to the Nir2 peptide. As shown in Figures 3b and 3c, in WT, for the residues directly contacting the peptide [12], [32], their HSQC peaks mostly disappeared at a molar ratio of 1∶2 (WT-MSP/Nir2), while for the residues not directly contacting but close to the binding interface, their HSQC peaks shifted (Figure 3c). Intriguingly, due to the reduced affinity, the addition of the Nir2 peptide only induced the shift, but not disappearance of the T46I HSQC peaks (Figure 3d).

### Binding interactions of WT-/T46I-MSP domains to EphA4

**Figure 4:**
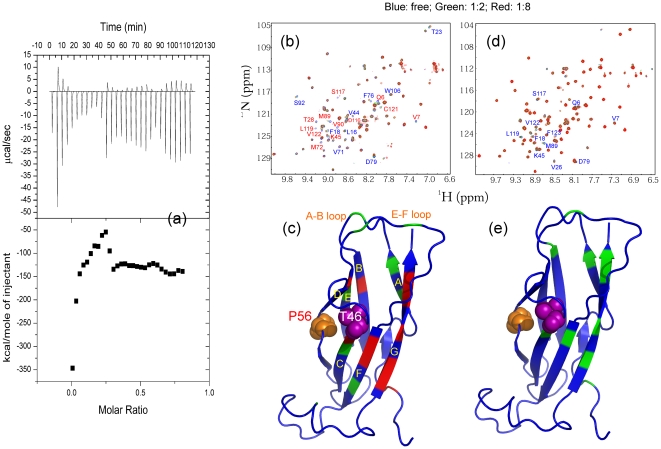
Interaction between the WT-/T46I-MSP domains and EphA4. (a). The ITC titration profile of the binding reaction of the WT-MSP domain to the ligand-binding domain of EphA4 (upper panel); and integrated values for reaction heats with subtraction of the corresponding blank results normalized by the amount of ligand injected versus molar ratio of MSP/EphA4 (lower panel). Superimposition of the two dimensional ^1^H-^15^N NMR HSQC spectra of the WT- (b) and T46I- (d) MSP domains in the absence (blue) and presence of the EphA4 ligand binding domain at molar ratios 1∶2 (green) and 1∶8 (red) (MSP/EphA4). Red letters are used to label residues with disappeared HSQC peaks while blue for residues with shifted HSQC peaks. The MSP structure with the perturbed residues mapped back for the interactions between the WT-MSP and EphA4 (c), and the T46I-MSP and EphA4 (e). Blue is to indicate residues with unperturbed HSQC peaks, while green and red for residues with shifted and disappeared HSQC peaks respectively.

Recently, the cleaved MSP domain was identified to serve as a novel ligand for the EphA4 ligand binding domain [29] but the underlying structural basis remains undefined. In the current study, we attempted to gain thermodynamic parameters for the EphA4-WT-MSP binding by ITC but unfortunately a complex ITC profile was obtain (Figure 4a), which implies that the binding may be involved in muti-sites or/and multi-steps. On the other hand, this profile was no longer observed for the T46I-EphA4 interaction [data not shown], implying that the T46I mutation significantly abolishes the binding.

Although previously we have successfully crystallized and determined the crystal structures of the WT-MSP domain [32] as well as EphA4 ligand binding domain in both free state [37] and in complex with ephrin-B2 [40], we failed to crystallize the EphA4-MSP complex after extensively screened a variety of buffer conditions. As such here we further gained the molecular details for the binding interaction between EphA4 and MSP by NMR HSQC titrations. First we monitored the change of the HSQC spectra of the ^15^N-labeled WT- and T46-MSP domains upon gradually adding the unlabeled EphA4 ligand binding domain. As shown in Figure 4b, introduction of EphA4 induced shifts of few peaks, but more dramatically disappearance of a set of peaks. If the residues with shifted and disappeared HSQC peaks were mapped back to the MSP structure, it appears that the significantly-perturbed residues are located on E-F loop and several β-strands: C-strand carrying Thr46, F- and G-strands (Figure 4c). By contrast, addition of EphA4 to T46I resulted in almost no disappearance the HSQC peaks, only caused slight peak shifts for some residues (Figures 4d and 4e). These results indicate that the T46I mutation dramatically disrupts the binding ability of the MSP domain to EphA4, nicely in agreement with the above ITC result.

**Figure 5:**
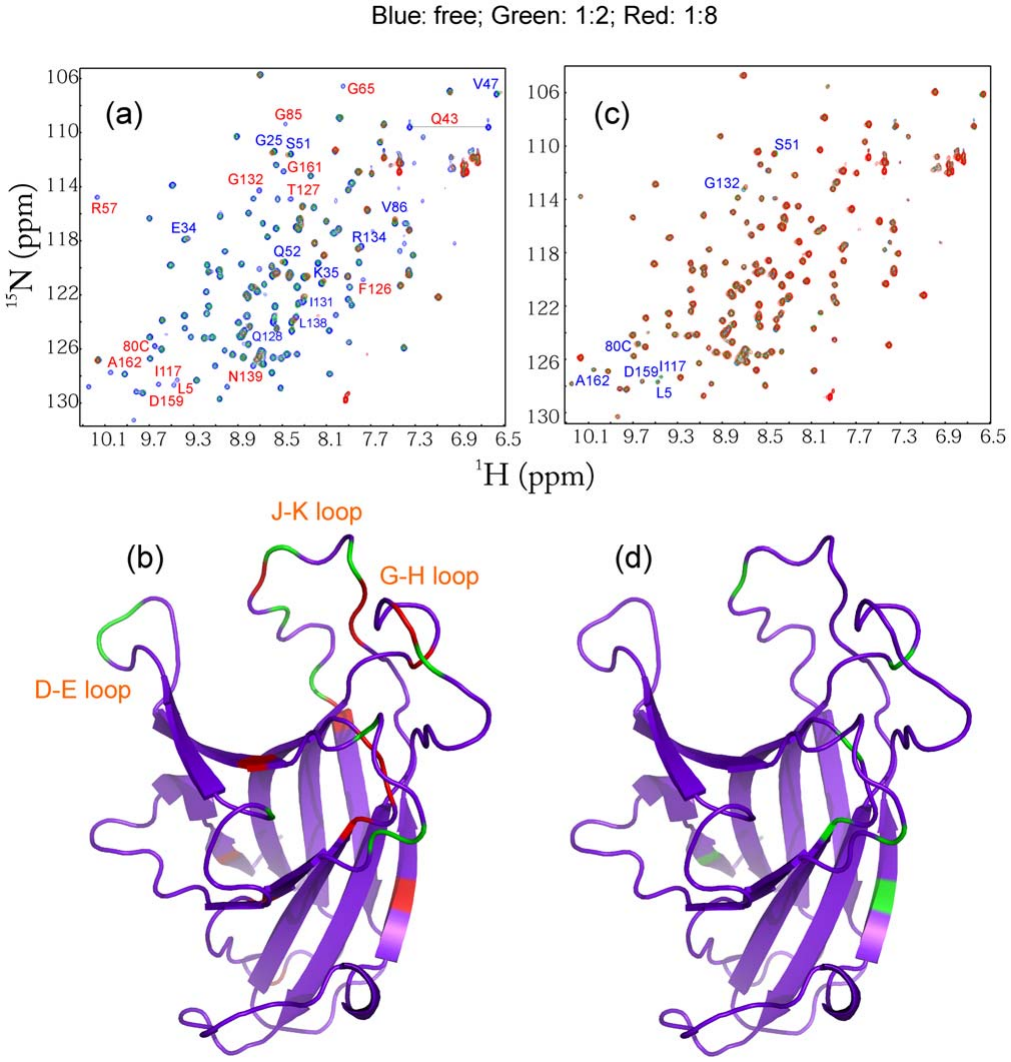
Interaction between EphA4 and WT-/T46I-MSP domain. Superimposition of the two-dimensional ^1^H-^15^N NMR HSQC spectra of the EphA4 ligand binding domain in the absence (blue) and presence of the WT- (a) and T46I- (c) MSP domains at molar ratios 1∶2 (green) and 1∶8 (red) (EphA4/MSP). Red letters are used to label residues with disappeared HSQC peaks while blue for residues with shifted peaks. Crystal structure of the EphA4 ligand-binding domain we previously determined (*37*) with the perturbed residues mapped back for the interaction between EphA4 and WT-MSP (b), and T46I-MSP (d). Blue is to indicate residues with unperturbed HSQC peaks, while green and red for residues with shifted and disappeared peaks respectively.

We further addressed the binding by monitoring the HSQC spectra of the EphA4 ligand-binding domain. As shown in Figure 5a, addition of the WT-MSP protein even at a ratio of 1:2 (EphA4/WT-MSP) triggered extensive disappearance or intensity-reduction of many EphA4 peaks, together with shifts of few peaks. If the ratio was increased up to 1∶8, most EphA4 peaks disappeared. By mapping the residues with shifted or disappeared peaks back to the EphA4 structure, it appears that the significantly affected residues are located over D–E, G–H and J–K loops, which have been previously established to directly contact it ligand ephrins [40]. By a sharp contrast, addition of the T46I-MSP domain caused almost no peak disappearance, but only shifts of few peaks (Figures 5c and 5d), again indicating that the T46I mutation dramatically eliminates the binding capability to EphA4.

To understand the underlying mechanism for the significant disappearance of both MSP and EphA4 HSQC peaks, we measured the radius of the EphA4 ligand binding domain by dynamic light scattering (DLS) in the presence of both WT- and T46I-MSP domains at different molar ratios but no significant changes were observed (data not shown). This implies that the binding triggers no formation of the tight EphA4-MSP complex and no significant oligmerization. As such, the disappearance of the MSP and EphA4 HSQC peaks is mostly resulting from the increase of conformational exchanges on the µs-ms time scale upon binding.

We also titrated the ^15^N-labeled EphA4 with the Nir2 peptide but no change was detected for the EphA4 HSQC spectra even at a ratio of 1∶10 (EphA4/Nir2), thus clearly demonstrating that no direct binding occurred between EphA4 and Nir2 peptide. Strikingly, in the presence of the Nir2 peptide, addition of the unlabeled WT-MSP domain was no longer able to induce shift/disappearance of the EphA4 HSQC peaks even with the ratio increased up to 1∶10 (EphA4/MSP). This observation strongly indicates that the binding of the Nir2 peptide to the MSP domain would inhibit its further binding to EphA4, either by triggering the structural change or occupying the EphA4 binding interface on the MSP domain. If considering the fact that the MSP domain show no significant change upon binding to ORP1, a FFAT-containing peptide [12], [32], the inhibition of the EphA4-MSP binding by the Nir2 peptide might largely result from the possibility that EphA4 and Nir2 peptide may have overlapped binding interfaces on the MSP domain.

**Figure 6:**
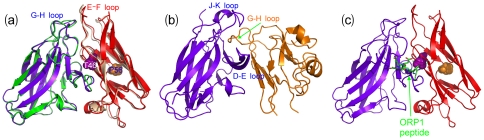
Docking model for the EphA4-MSP complex. (a). Superimposition of two lowest energy docking model of the EphA4-MSP complex. (b). The crystal structure we previously determined for the EphA4-ephrinB2 complex [40]. (c). The docking model of the EphA4-MSP complex with the FFAT-containing ORP1 peptide displayed according to the VAPA MSP-ORP1 structure [12].

Based on the HSQC titration results, we also constructed the model of the EphA4-MSP complex by HADDOCK and Figure 6a presents the two lowest-energy models. Interestingly, although the overall architecture of the EphA4-MSP complex bears some similarity to the EphA4-ephrinB2 complex [40], some fundamental differences were observed. As previously uncovered, in all Eph-ephrin complexes, the ephrin G-H loop inserts into the hydrophobic ligand-binding channel of Eph receptor constituted by the convex sheet of four β-strands together with the D-E, J-K, and G-H loops (Figure 6b). Previously, by using NMR HSQC titration, we showed that the EphA4 residues on the convex sheets either disappeared or significantly shifted upon binding to ephrinB2 [40]. However, in the EphA4-MSP complex there is no MSP residues inserting into the EphA4 channel. Instead, the MSP E–F loop is found to interact with EphA4 loops including G–H and J–K loops (Figure 6a). This property is strongly supported by our NMR results that no significant perturbation was observed on the residues on the EphA4 convex sheets upon binding to the MSP domain. From this model, binding residues on both MSP and EphA4 are mostly located on loops which are thus highly dynamic. As a consequence, the binding affinity between EphA4 and MSP is expected to be relatively-weak, which is consistent with ITC and NMR results.

Also in the EphA4-MSP model, Thr46 sits in the central part of the EphA4-MSP binding interface (Figure 6a), which rationalizes the experimental observation that the mutation of Thr46 to Ile dramatically abolishes its binding ability to EphA4. On the other hand, in this model EphA4 and FFAT-containing peptide indeed have overlapped binding interfaces on the MSP domain (Figure 6c), thus nicely explaining another experimental observation that the binding of the Nir2 peptide to the MSP domain will inhibit its further interaction with EphA4.

### Molecular dynamics (MD) simulations of the WT-/T46I-MSP domains

Our results reveal that the T46I mutation causes no significant disruption of the native MSP structure, but it does lead to ALS. This implies that the protein dynamics may play a key role beyond the static structure, as we recently demonstrated on the SARS 3C-like protease [36]. As such here we utilized the molecular dynamics (MD) simulation to explore the dynamical behavior of the MSP domain as well as the consequence of the T46I mutation.

**Figure 7:**
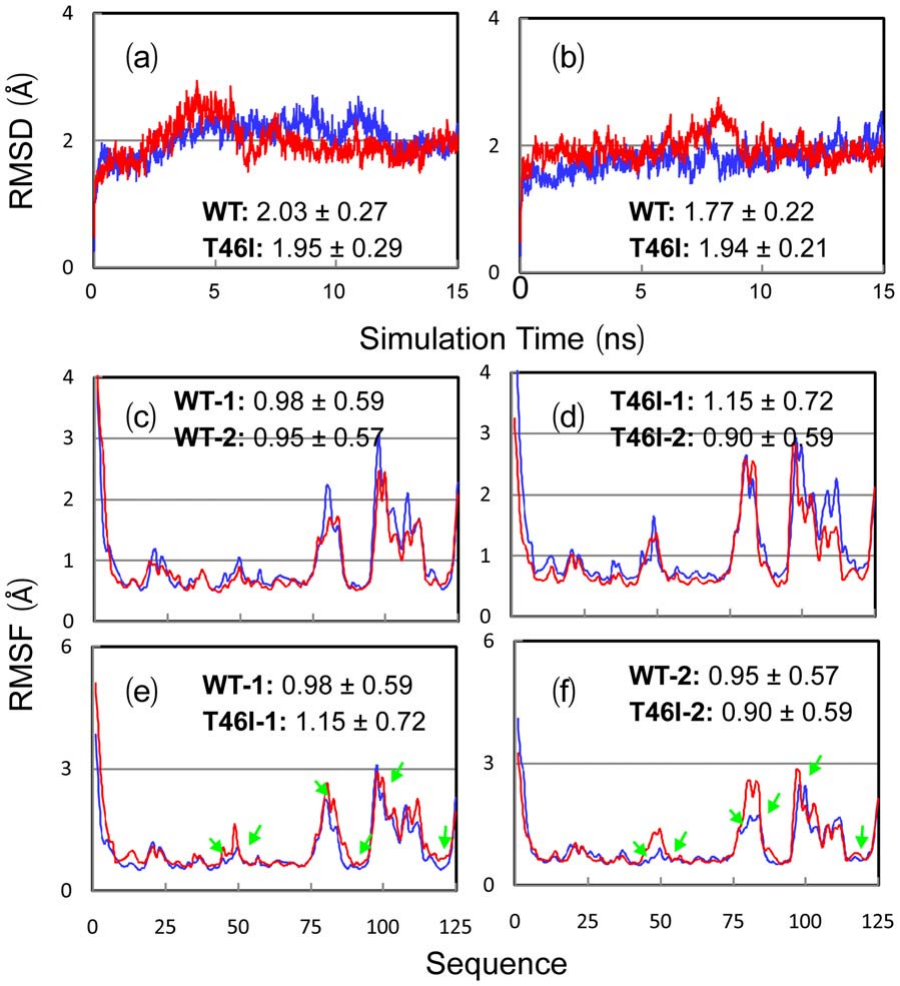
Trajectories of MD simulations. (a–b). Root-mean-square deviations (RMSD) of the backbone atoms for two independent MD simulations of the WT- (blue) and T46I- (red) MSP domains. (c–d). Root-mean-square fluctuations (RMSF) of the Cα atoms computed for two independent simulations (blue for simulation 1 and red for simulation 2) of the WT- (c) and T46I- (d) MSP domains. (e–f). Root-mean-square fluctuations (RMSF) of the Cα atoms computed for two independent simulations for the WT- (blue) and T46I- (red) MSP domains. The average values and standard deviations over 15 ns are computed and displayed. The green arrows are used to indicate the T46I-MSP residues with fluctuations larger than those of the WT-MSP domain.

Figures 7a and 7b present the root-mean-square deviations (RMSD) of backbone atoms for two parallel simulations for both WT- and T46I-MSP domains. It appears that for all simulations, the RMS deviation values increased very rapidly during the first 0.1 ns. This is mostly due to the relaxations of the crystal structures upon being solvated in solution. Surprisingly, the WT- and T46I-MSP domains display no significant difference for overall dynamical behaviors, as judged from their similar trajectories and average RMSD values.

**Figure 8:**
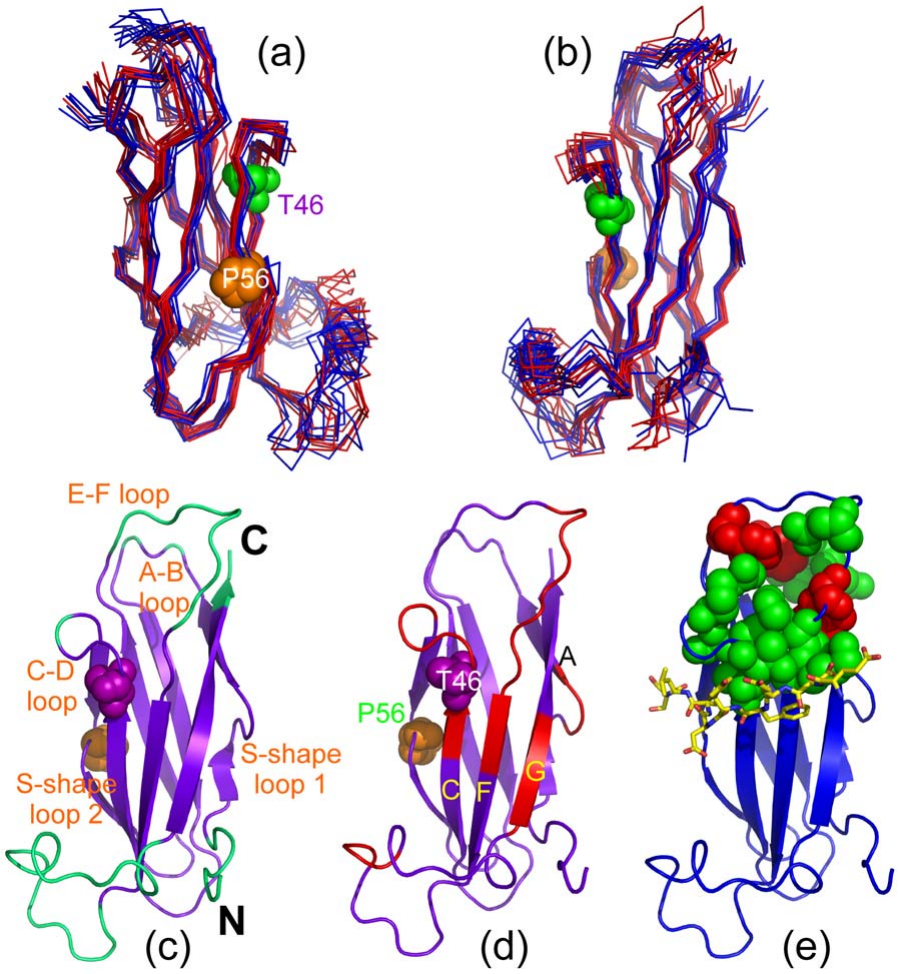
Dynamical behaviors. (a–b). Structure snapshots (one structure for 1-ns interval) for two independent MD simulations for the WT- (blue) and T46I- (red) MSP domains. (c). The MSP structure in which cyan is utilized to indicate the WT-MSP residues with RMSF values larger than the average. (d). The MSP structure in which red is used to indicate T46I residues whose RMSF values are larger than those of the WT-MSP domain. The two mutation residues causing ALS, P56S and T46I, were displayed as spheres. (e). The MSP structure in which green is used to indicate residues whose hydrogen bonds occupancy difference is larger than 10% between WT and T46I while red is for residues whose hydrogen bonds occupancy difference is larger than 10% between T46I and WT.

Snapshots of two independent simulations are presented in Figures 8a and 8b respectively for the WT- and T46I-MSP domains. As shown in Figures 7c and 7d which present the root-mean-square fluctuation (RMSF) values versus the sequences, although the global patterns of two independent simulations for either WT or T46I are similar, there do exist some significant differences over some local regions. This is due to the fact that the systems behave non-ergodic in 15-ns simulations and as a consequence the results would be influenced by the initial conditions such as velocities. Interestingly, as seen in Figures 7e and 7f, the WT-MSP residues with the RMSF value > the average value include N-terminal Met1-Val7, Pro21 and Thr23 on A–B loop, Arg50 on C–D loop, Phe76-Ly85 on E-F loop, Pro96-Leu114 containing the unique helical fragment, and C-terminal Phe123-Leu125 (Figure 8c). These residues were also found to be highly dynamic on the min-hr time scale as captured by the H/D exchange experiments.

Overall, the T46I-MSP domain has fluctuation patterns similar to those of the WT-MSP domain (Figures 7c and 7d). Nevertheless, increases of local fluctuations do occur for the T46I-MSP domain. For example, in both simulations (Figures 7c and 7d), several T46I regions show increased dynamics, which include Gln13-Glu15 on the first proline-constrained S-shape loop [32], Val44-Arg50 on C-strand and C-D loop covering the mutation site Thr46; Pro80-Val90 on E-F loop and F-strand; Glu103-Val105 on the helical fragment, and Met 115-Lys118 on the G-strand (Figure 8d). On the other hand, we also calculated the hydrogen bond occupancy for the WT and T46I simulations and surprisingly only a small number of hydrogen bonds have the occupancy difference larger than 10% between the WT- and T46I-domains (Table 1). Nevertheless, residues with higher fluctuations (Figure 8d) and significantly-reduced hydrogen bond occupancy in T46I (Figure 8e) also overlap with those experimentally mapped to be critical for binding to EphA4 and Nir2 peptide (Figures 4 and 5). As such, the simulation results suggest that the dynamical changes triggered by the T46I mutation may at least partly account for the loss of the binding affinity to EphA4 and Nir2 peptide.

**Table 1:** Hydrogen Bond Occupancy in MD simulations for the WT- and T46I-MSP Domains.

Residue	Residue Type	Atom Type	Residue	Residue Type	Atom Type	Average^a^ [WT]	Average^a^ [T46I]	Difference^b^
19	ARG	NE	124	GLU	OE2	19.98	6.32	13.66
46	THR	OG1	52	TYR	O	27.84	0.00	27.84
46	THR	OG1	87	LYS	O	34.01	0.00	34.01
47	THR	N	87	LYS	O	86.24	64.35	21.89
51	ARG	NH1	82	GLU	OE1	14.91	3.26	11.65
51	ARG	NH1	82	GLU	OE2	17.28	1.76	15.52
51	ARG	NH1	86	HIS	NE2	55.69	13.35	42.34
76	PHE	N	22	PHE	O	29.82	49.10	−19.27
84	SER	OG	121	CYS	O	3.90	24.24	−20.34

a. The displayed values of the hydrogen bond occupancy are averaged over two independent simulations with 15 ns for WT- [wild-type] and T46I-MSP domains respectively.

b. Only hydrogen bonds with the occupancy difference between WT and T46I >10% are displayed in this table.

## Discussion

Last year, from non-Brazilian kindred the second mutation T46I has been identified on the hVAPB MSP domain which also causes a familial amyotrophic lateral sclerosis [6]. However, so far, it remains completely unknown as how this mutation affects the structure, stability, dynamics and binding function of the MSP domain. In the present study, we aimed to address these issues by utilizing both experimental and computational approaches. Unlike the P56S-MSP domain with the entirely eliminated native structure and binding capacity, the T46I-MSP domain still retains the native secondary and tertiary structures, as evidently shown by extensive CD and NMR characterization. Therefore, totally different from P56S, T46I forms another class of the ALS-causing MSP mutations which are analogous to some SOD1 mutants with the native three-dimensional structure preserved [53], [54].

Nevertheless, the T46I mutation does abolish the cooperative urea-unfolding transition and reduce the thermodynamic stability of the MSP domain. This allows the T46-MSP domain to more easily access the partially- or/and highly-unfolded intermediates which are prone to aggregation [32]–[35]. Indeed, if compared to the WT-MSP domain, the T46I-MSP domain is much more prone to aggregation at high protein concentration and temperature. Moreover, as in cells macromolecular solutes are expected to reach concentrations of >300 g/L and occupy >30% of the volume [48], [55]–[58], the tendency of the T46I mutant to aggregate in cells should become more severe. It was once believed that in macromolecular crowded cellular environments, the native state should be stabilized by excluded-volume effects because it occupies less space than the denatured state [48], [55]. However, recent studies of protein stability in cells uncover a surprising result; proteins are either unaffected or destabilized in cells [56]–[58]. Now it is proposed that the destabilization arises from a competition between stabilizing excluded-volume effects and destabilizing nonspecific interactions, in particular electrostatic interactions which become predominant in the crowded cellular environment [56]–[59]. As such, although the T46I-MSP domain still preserves the native MSP structure *in vitro*, the T46I-triggered loss of the cooperative unfolding and stability may be sufficient to cause severe aggregation in macromolecular crowded cells, thus rationalizing the previous *in vivo* observation [6].

Amyotrophic lateral sclerosis (ALS) was described more than 130 years ago, but it still remains a mystery whether any signaling pathway is involved in ALS development. In this regard, characterization of ALS-caustive mutations on the VAPB MSP domain offers an unprecedented opportunity because two signaling networks have been identified to be associated with the VAPB MSP domain; one linked to lipid trafficking/metabolism via binding to the FFAT-containing proteins; and another connected to Eph-ephrin signaling, by acting as a novel ligand for Eph receptors. Here we show that the T46I mutation results in only a 3-fold binding affinity reduction to the Nir2 peptide but dramatically disruption of the binding capability to EphA4. In particular, we reveal that the Nir2 peptide and EphA4 are most likely to have overlapped binding interfaces on the MSP domain and consequently the binding of the Nir2 peptide prevents the MSP domain from further interacting with EphA4. This thus raises an interesting possibility that the two signaling networks may interplay *in vivo*. As a consequence, future studies on how they interplay *in vivo* under physiological and ALS pathological conditions may shed critical light on ALS pathogenesis.

With significant advances in experimental and computational methodologies including NMR spectroscopy and MD simulations, protein dynamics have been recently demonstrated to play a key role in functions [36], [60], [61]. Here we also experimentally and computationally explored the dynamical behaviors of both WT- and T46I-MSP domains. Our H/D exchange results indicate that even the WT-MSP domain is very dynamic on the min-hr time scale, with fast-exchange rate residues located not only on most loop regions, but also extensively over the helix and β-strands. Furthermore, MD simulations unravel that although the T46I mutation leads to no significant alteration of the overall dynamics, it does trigger increases of fluctuations over several local regions. Most amazingly, the residues with enhanced dynamics appear to be involved in binding to both Nir2 peptide and EphA4. As such, the T46I-triggered enhancement of local dynamics may also contribute to the observed disruption of the binding capacity of the MSP domain.

In conclusion, here we reveal that unlike the P56S mutation which completely eliminates the native MSP structure, the T46I mutation results in no dramatic structural disruption and alteration of overall dynamics. Nevertheless, the T46I mutation does leads to the loss of the cooperative unfolding transition and reduction of thermodynamic stability, which consequently renders the T46I-MSP domain to be prone to aggregation at high protein concentrations and temperatures. Furthermore, the T46I mutation leads to only a 3-fold affinity reduction to the Nir2 peptide but remarkable loss in binding to EphA4. In particular, the overlapped interfaces on the MSP domain for binding to EphA4 and Nir2 peptide strongly implies that two signaling networks may functionally interplay *in vivo*. Future understanding how the interplay alters under ALS pathological conditions may decipher the mystery of ALS pathogenesis.
